# Benefits from Implementing Low- to High-Intensity Inspiratory Muscle Training in Patients Undergoing Cardiac Surgery: A Systematic Review

**DOI:** 10.3390/jcdd11120380

**Published:** 2024-11-27

**Authors:** Aphrodite Evangelodimou, Irini Patsaki, Alexandros Andrikopoulos, Foteini Chatzivasiloglou, Stavros Dimopoulos

**Affiliations:** 1 Laboratory of Advanced Physiotherapy, Physiotherapy Department, School of Health & Care Sciences, University of West Attica, 12243 Athens, Greece; aevangelodimou@uniwa.gr (A.E.); epatsaki@uniwa.gr (I.P.); 2 Clinical Ergospirometry, Exercise and Rehabilitation Laboratory, 1st Critical Care Department, Evangelismos Hospital, School of Medicine, National and Kapodistrian University of Athens, 10675 Athens, Greece; alexandrikopoulos@gmail.com; 3 Intensive Care Unit, Bristol Royal Infirmary University Hospitals Bristol and Weston NHS Foundation Trust, Bristol BS2 8HW, UK; fay.tza@gmail.com; 4 Cardiac Surgery ICU, Onassis Cardiac Surgery Center, Kallithea, 17674 Athens, Greece

**Keywords:** inspiratory muscle training, cardiac surgery, coronary artery bypass

## Abstract

Cardiac surgery procedures are among the main treatments for people with cardiovascular disease, with physiotherapy playing a vital part. Respiratory complications are common and associated with prolonged Intensive Care Unit (ICU) and hospital stay, as well as increased mortality. Inspiratory muscle training has been found to be beneficial in improving respiratory muscle function in critically ill patients and patients with heart failure. The purpose of this review is to present the results of implementing inspiratory muscle training (IMT) programs in patients before and/or after cardiac surgery. The PubMed, Embase and Science Direct databases were searched from January 2012 to August 2023. In the present review, randomized controlled clinical trials (RCTs), clinical trials and quasi-experimental studies conducted in adult patients pre and/or post cardiac surgery were included. Fifteen studies were considered eligible for inclusion in the review. The results revealed that the IMT programs varied in intensity, repetitions, and duration in all included studies. Most studies implemented the IMT after the surgery. Statistical significance between groups was noted in Maximal Inspiratory Pressure and the 6-Minute Walk Distance Test. Preoperative and postoperative programs could improve inspiratory muscle strength, pulmonary function, and functional capacity as well as decrease the length of hospital stay in patients undergoing cardiac surgery. No clear evidence emerged favoring low or higher IMT intensities. The combination of IMT with other forms of exercise might be beneficial in patients undergoing cardiac surgery. However, further RCTs are required to provide confirming evidence.

## 1. Introduction

Over the past few years cardiovascular diseases have been recognized as a significant health problem with increased morbidity [1]. Cardiac surgery procedures are among the main treatments for them, with physiotherapy playing a vital part prior and after the procedure [2]. Early mobilization and respiratory physiotherapy have been widely implemented to prevent complications and augment recovery in these patients. Respiratory complications are common and are associated with prolonged Intensive Care Unit (ICU) and hospital stay, as well as increased mortality [3]. In particular, preoperative chest physiotherapy seems to have a protective role against the development of pulmonary complications [4]. Inspiratory muscle training has been found to be beneficial in improving respiratory muscle function in critically ill patients [5] and patients with heart failure [6]. Technological innovation has pushed the boundaries of rehabilitation by introducing new means and devices that could offer an advanced program better tailored to the needs of the patient. The aim of this study was to carefully review and present not only the results of implementing threshold Inspiratory Muscle Training (IMT) in patients pre or/and post cardiac surgery but to present all programs that have been described in studies published in the last decade as well.

## 2. Materials and Methods

### 2.1. Study Design

The present study was conducted following the Preferred Reporting Items for Systematic Review and Meta-Analyses (PRISMA) guidelines [7] and the methodological quality assessment of the clinical trials was conducted according to the PEDro scale [8].

### 2.2. Data Sources and Search Strategies

A literature search was performed in the PubMed, Science Direct and Scopus online databases for manuscripts published from January 2012 to 30 August 2023, using the following keywords both alone and in different combinations in order to create different search strategies: “inspiratory muscle training”, “inspiratory muscle exercise”, “respiratory muscle training”, “cardiac surgery”, “coronary artery bypass”, “strength”, “dyspnea”, “functional capacity”, “pulmonary function” and “hospitalization length”. The search was limited to the English language. The search was also extended to studies cited as references in relevant research articles. The search strategy was designed and conducted by two independent reviewers.

### 2.3. Eligibility Criteria

The PICO framework was utilized to form the eligibility criteria of the review, as recommended by Cochrane [9]. The framework focuses on the population (cardiac surgery patients), intervention (inspiratory muscle training), comparison and outcomes of the studies (pulmonary function, capacity, and strength). It is established as a commonly used tool for quantitative systematic reviews by both Cochrane Collaboration and the National Library of Medicine [10].

The studies included in the systematic review met the following criteria: randomized controlled clinical trials, clinical trials or quasi-experimental studies, published in the English language, conducted in the last 11 years, from January 2012 to 30 August 2023; an adult population with no sex limitation; and in-hospital, community-based or home-based IMT programs in patients undergoing cardiac surgery. The studies should include preoperative or postoperative IMT programs alone or combined with other exercise training programs. The outcomes should include inspiratory muscle strength, pulmonary function, functional capacity, pulmonary complications, quality of life and hospital stay.

Studies on pediatric populations, non-human studies, abstracts, dissertations and case reports were excluded. Studies with no information on clinical outcomes were also excluded.

### 2.4. Data Extraction

All study titles and abstracts based on the searched electronic results were screened by two independent reviewers. The two reviewers also assessed all full texts of potentially eligible studies to select those that met the inclusion criteria. The possible discrepancies between the reviewers were resolved by another senior reviewer to reach the final decision by consensus.

#### The Data Extraction Was Based on PICO Framework

Population: The studies included in the review conducted interventions in adults undergoing any type of cardiac surgery. The data extracted concerning the participants were age, sex, and the type of cardiac surgery.

Intervention: The intervention included inspiratory muscle training programs in the preoperative and postoperative period. The data extracted concerning the intervention included the type of the device, the resistance inspiratory load, the repetitions and the duration of the inspiratory muscle training program and the interventions in the control group of patients.

Outcome measures: The primary outcome measure was the inspiratory muscle strength assessed by measuring the maximum inspiratory pressure (MIP). The secondary outcomes included pulmonary function, functional capacity and hospital stay.

### 2.5. Quality Assessment

A quality assessment of all selected studies was performed by two independent authors who used the PEDro scale [8]. This scale is recognized as a useful tool for the evaluation of the quality of clinical trials and consists of 11 items: 1. specified eligibility criteria; 2. subjects’ random allocation to groups; 3. allocation concealed; 4. baseline comparison; 5. The blinding of all subjects; 6. the blinding of therapists; 7. the blinding of the assessors; 8. at least one key outcome was obtained from more than 85% of the subjects initially allocated to groups; 9. intention-to-treat analysis; 10. between-group statistical comparisons; and 11. point measures and measures of variability. The items are scored as present (1) or absent (0). The obtained score of each study ranges from 0 to 10, as the first item is not used to calculate the final score [8].

## 3. Results

### 3.1. Selection and Study Characteristics

**Figure 1:**
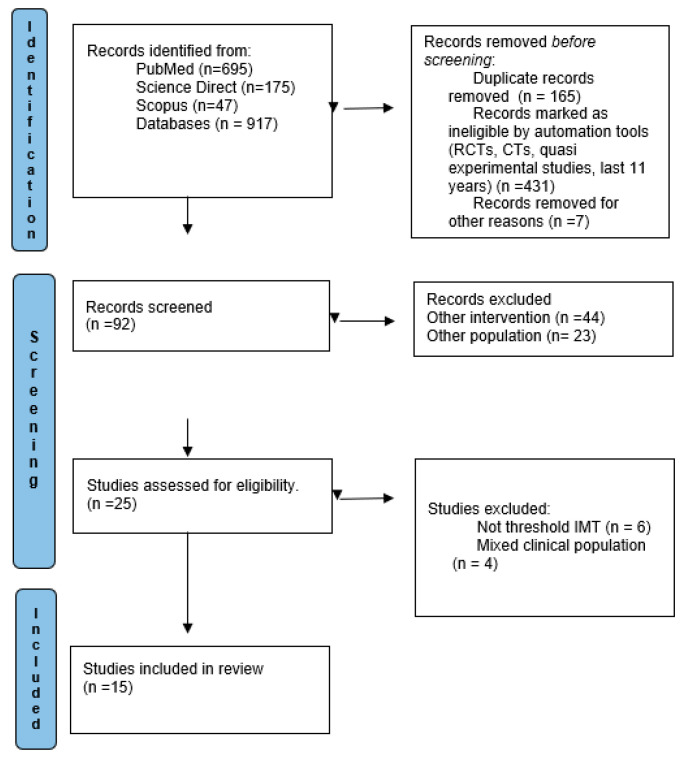
Flow diagram.

The online database search originally identified 917 articles. A review of all studies of the last decade was performed after the removal of duplicates, and 15 studies [11,12,13,14,15,16,17,18,19,20,21,22,23,24,25] were considered eligible for inclusion in the review. The specific study selection flow chart is presented in Figure 1.

All studies were either randomized control clinical trials, clinical trials, or quasi-experimental studies. The main characteristics of the studies are presented in Table 1.

### 3.2. Methodological Quality

The evaluation of the methodological quality with the PEDro scale was conducted by the reviewers. The score of the articles ranged from 4 to 8, with a mean score of 5.7 (Table 2). Overall, eight studies [11,13,15,16,17,18,23,24] were assessed methodologically as fair (having a score of 4–5) and seven studies [12,14,19,20,21,22,25] as good (having a score of 6–8). Item 6 concerning the blinding of the therapist was absent in all studies. The blinding of the patients (item 5) was present only in three studies.

### 3.3. Characteristics of Included Studies

The 15 studies included in the review implemented IMT before and/or after cardiac surgery (coronary artery bypass graft surgery—CABG, heart valve replacement). Most of the studies [11,12,14,15,17,18,19,20,21] applied IMT postoperatively, two studies [13,22] applied IMT preoperatively and one study [16] applied IMT both preoperatively and postoperatively.

The population participating in the studies was a total of 815 patients, with 57.92 ± 7.84 (mean ± SD) years of age in the intervention groups and 58.9 ± 8.39 years (mean ± SD) in the control groups. In the studies included, 555 patients underwent coronary artery bypass surgery, 195 patients heart valve replacement surgery, 23 patients both coronary artery bypass graft (CABG)–valve replacement and 31 patients atrial septal defect correction. Two studies included only male patients [16,17].

### 3.4. IMT Programs

The implemented IMT programs in the intervention groups varied in intensity, repetitions, and total duration. All studies used a resistive device for inspiratory muscle training. The device was described as a linear pressure loading device in the studies of Cordeiro et al. [15], Miozzo et al. [18], Cargnin et al. [21] and Cordeiro et al. [23]. The other studies [11,12,13,14,16,17,19,20,22,25] described the device as an inspiratory threshold loading device or trainer, and only Fortes et al. [24] referred to the device only as an electronic device.

Intensity: The baseline intensity of the IMT load was determined before the training program. The inspiratory pressure load was readjusted during the training in all studies, except for Sobrinho et al. [13], who used a constant load of 40% of MIP in their preoperative training. The initial intensity varied from 20% of MIP [19] to 30% of MIP [14,16,21,22,24] and 40% [11,13,15]. The intensity of the inspiratory pressure was readjusted in the studies according to the perceived exertion using the Borg Scale and escalated up to 80% of MIP. When the score of the perceived exertion was less than five in the scale, the intensity increased.

Kodric at al. [12] implemented a program with four different intensities at 30%, 70%, 15% and 80% of MIP applied for 5 min each. Elmarakby et al. [17] set the initial intensity at 30%, which increased up to 60 to 80% until discharge. Two studies [18,20] initiated training in higher intensity at 50% of MIP up to 80% at the end of the program. The intensity of IMT in the Cordeiro et al. [23] study was based on anaerobic threshold and varied from 10% of MIP up to 80% of MIP, according to the patient’s performance. Patients in the Hegazy et al. [25] study started training at 40% of MIP, which increased from 5% to 10% according to the patient’s tolerance, targeting 80% of MIP at the end of the program.

Repetitions and duration: The repetitions of the IMT program were applied variously in the studies. Five studies [11,13,14,15,16] implemented the IMT program in three sets of 10 repetitions of breaths once or twice daily. Two studies [18,20] implemented the program in five sets of 10 repetitions and one [19] in eight sets of 10 repetitions once a day. Cordeiro et al. [23] implemented the IMT program for 15 repetitions in each load, while in three studies [17,21,24], the training lasted for 30 breaths.

The session time of the IMT program reported in some studies was mostly consistent at 20–30 min [12,22,25]. Chen et al. [22] and Hegazy et al. [25] applied the IMT program twice a day.

The total duration of the IMT program when applied postoperatively varied and lasted from 3 days [11] to 12 weeks [14,18]. Kodric et al. [12] implemented the program for 12 months, but the patients enrolled in the study were diagnosed with diaphragmatic paralysis due to phrenic nerve injury. In some studies, the total duration was not stated. In both studies by Cordeiro et al. [15,23], the IMT program was applied until the day of hospital discharge. But in Elmarakby et al. [17], the duration was not specified.

In some studies, IMT programs were combined with other interventions. Miozzo et al. [18] combined IMT with aerobic exercise, Hermes et al. [14] with cardiac rehabilitation program, dos Santos et al. [20] with resistance and aerobic training and Zanini et al. [19] combined the IMT with chest physiotherapy and exercise in the first intervention group and with chest physiotherapy alone in the second intervention group.

Control group interventions: The control group received various interventions in the included studies. The combination of chest physiotherapy and mobilization was applied in five studies [11,16,19,24,25]. Chest physiotherapy was implemented in two studies [17,19]. In one study [14], the control group participated in a cardiac rehabilitation program. However, there were two studies [13,15] stating that the control group received the usual care. Sham IMT was delivered in the control group in four studies [12,20,21,22], with a minimum load of 9 cm H2O or 9% of MIP. In the study of Cordeiro et al. [23], the control group received an IMT program at 40% of MIP for three sets of 15 repetitions.

### 3.5. Outcome Measures

Inspiratory muscle strength was assessed by measuring the MIP in the studies included. The procedure was described in all studies, and eight studies [12,14,15,16,17,21,22,25] reported a significant increase (*p* < 0.05) in MIP in comparison to the control group, even in studies applying training after the surgery.

Pulmonary function was assessed by measuring Vital Capacity (VC), Forced Vital Capacity (FVC), Forced Expiratory Volume in the first second (FEV_1_) and FEV_1_/FVC in six studies [11,12,19,21,22,25]. All studies implemented IMT after surgery. One study [11] reported a significant decrease (*p* < 0.05) in VC after surgery and four studies reported a significant increase in FEV_1_ in the intervention group [12,19,22,25], with three [12,19,25] of them reporting a remarkable difference (*p* < 0.001) in comparison with the control group.

Two studies [16,17] measured the A-a gradient, which provides a simple method of assessing alveolar–capillary gas exchange. Both studies reported a significant difference in this parameter in the IMT group after CABG. Interesting in both studies, the intervention started before the surgery and continued after it.

Functional capacity was assessed with the 6-Minute Walking Distance Test (6MWDT) in six studies [15,19,20,21,23,25], and five [15,19,20,21,23] of them reported a significant increase (*p* < 0.05) in the intervention group that reached significant statistical difference between groups (*p* < 0.05).

Length of hospital stay: three studies included in the review reported a significantly shorter length of hospital stay in patients following an IMT program [13,22,23].

## 4. Discussion

The purpose of this systematic review was not only to investigate the effectiveness of IMT in patients undergoing cardiac surgery but also to assess, from a clinical perspective, the training programs that were being implemented. Thus, novel information is presented to augment the efficiency of this therapeutic intervention. The results suggest that cardiac surgery patients could have a significant improvement in inspiratory muscle strength, functional capacity, and lung function following IMT. These findings were also underlined by previously published systematic reviews and meta-analyses [26,27,28] that investigated the effect of the pre- and post-surgery implementation of IMT. However, their research was limited up to 2021 and included a more specific population [28], and fewer studies were included compared to the present study. In particular, Zhang et al. included studies up to 2017, although their research continued up to December 2021. Additionally, the authors included other respiratory muscle trainers besides thresholds, like a flow-dependent and expiratory positive airway pressure mask. As pulmonary complications prolong postoperative ICU and hospital stay, and are consequently being associated with increased hospital costs [29], the probability of further complications [30] and an increased risk of mortality, it is essential to recognize interventions that could minimize all these risks. It is well documented that patients undergoing cardiac surgery may already have reduced respiratory muscle strength [31]; however, after surgery, this is more evident [12]. Furthermore, respiratory dysfunction preoperatively leads to reduced functional capacity postoperatively [32,33]. Therefore, it is surprising that most studies during the last 10 years implement IMT only post cardiac surgery. To the best of our knowledge, only Turky et al. [16] and Elmarakby et al. [17] gave importance to a program that starts before surgery and continues after it. Hegazy et al. [25] noted that the intervention group showed the gradual recovery of MIP after the first postoperative week and was closer to the preoperative level by the end of the first postoperative month. Moreover, the authors noted that by the end of the eighth postoperative week, the experimental group presented a significant increase in MIP, which was much higher than the preoperative level and higher than the control group as well. In clinical practice, patients with reduced MIP preoperatively should follow an IMT program, even when following a cardiac rehabilitation program is not applicable. The beneficial effect of IMT is consistent in both low and high loading. When selecting the appropriate intensity, we should take into consideration co-morbidities such as chronic obstructive pulmonary disease and what will be the target of the training strength or endurance. We should also consider the characteristics of the exercise program that the patient follows postoperatively in order not to overload the patients. In any case, high-intensity loading was found to be safe even in this fragile population, and it was noted that they were able to reach a higher improvement in MIP [25]. However, in most studies, high intensities were achieved incrementally over the whole training period.

Although the assessment of functional capacity is an important indicator of physical performance and quality of life, commenting on minimal clinical importance difference (MCID) could be more useful. Thus, just showing a significant change in 6MWDT may not be adequate if this change does not reach MCID [34]. Moreover, 6MWDT was reported to reach MCID in two studies [19,25]. It is often noted that there is a decrease in the distance covered during 6MWDT following cardiac surgery, yet in the study of Cordeiro et al. [15], there was no difference in the distance covered by the IMT group between the preoperative period and at discharge, suggesting the maintenance of functional capacity. Furthermore, it is important to underline that changes were able to be maintained, even after the completion of the rehabilitation program [25]. An improvement in functional capacity is anticipated following a rehabilitation program, as seen in other chronic diseases such as chronic obstructive pulmonary disease (COPD) [33].

IMT has been found to be beneficial, even in reducing hospital length of stay [15,22]. Minimizing exposure to risk factors related to hospital stay is of high importance in patients undergoing cardiac surgery [35]. Additionally, it should be underlined that length of stay is an independent factor for functional limitation [36].

There have been different inspiratory training methods applied, but due to the heterogeneity of the studies included in our systematic review, we are unable to provide evidence for greater beneficial effects of high- versus low-intensity IMT methods, both being safe and efficient. Similar inconclusive results have been recently found by Patsaki et al. [37] in a meta-analysis performed regarding differences between low–medium- and high-intensity IMT in patients undergoing mechanical ventilation in critically ill patients.

The positive findings of this systematic review are in accordance with previous ones, limited to fewer and older studies that implemented IMT preoperatively [38], post [39] or during both [27] phases. A recent meta-analysis investigating IMT in patients with CABG has provided similar findings [40], while another one has recently shown that both IMT and expiratory muscle training improves respiratory muscle strength; however, there was no evidence indicating the efficacy of IMT for pulmonary function and length of hospital stay and the efficacy of EMT for functional capacity [41].

This systematic review has some limitations: the total sample size was small, and there was little participation of elderly patients. Comorbidities that affect MIP (heart failure, COPD, and chronic renal failure) might have influenced the study results and should be taken into consideration in future studies. Nutritional interventions could also play a vital role in this population as preoperative high fat mass and low free fat mass content are independent predictors of prolonged hospital length of stay [42]. Malnutrition has been related to a higher prevalence of comorbidities and mortality [43]. IMT prescription was not identical in all studies. Only two studies implemented IMT before surgery, limiting definite conclusions in potential additional benefits by this practice; further studies are needed.

## 5. Conclusions

Inspiratory muscle training improves inspiratory muscle strength, functional capacity, and pulmonary function and may reduce ICU length of stay in patients undergoing cardiac surgery. There is no clear evidence favoring low or higher IMT intensities. The combination of IMT with other forms of exercise seems beneficial, yet we need further RCT studies to provide more evidence.

## Figures and Tables

**Table 1:** Main PICOS characteristics of the studies included in the systematic review.

Study (Year)	Procedures–Patients (N)	Intervention Group	Control Group	Outcomes	Results
Matheus et al., 2012 [11] Brazil	After CABG IG:23, CG:24	40% MIP, 10 rep, twice/day for 3 days	Incentive spirometry, ambulation	MIP, MEP, TV, VC	TV and VC on 3rd postoperative day (*p* < 0.05)
Kodric et al., 2013 [12] Italy	Diaphragm paralysis after CABG and valve replacement IG:46, CG:23	5′ at 30% MIP 5′ at 70% MIP 5′ at 15% MIP 5′ at 80% MIP, daily	Sham IMT	MIP, MEP, FEV_1_/VC, TLC MRC dyspnea scale	MIP *p* < 0.05 at 3, 6 and 12 months MRC: *p* < 0.05
Sobrinho et al., 2014 [13] Brazil	Before CABG IG:35, CG:35	40% MIP, 3 set/10 rep, once/day	Usual care	MIP, MEP, MV	NS
Hermes et al., 2015 [14] Brazil	After CABG IG:12, CG:12	30% MIP, 3 set/10 rep	Cardiac rehab program	MIP, MEP, VO_2_ peak	*p* < 0.001
Cordeiro et al., 2016 [15] Brazil	After heart surgery (CABG, Valve) IG:25, CG:25	40% MIP, 3 set/10 rep, twice/day until hospital discharge	Usual care	MIP, 6MWDT	MIP and 6MWDT *p* < 0.05
Turky et al., 2017 [16] Egypt	Before–After CABG IG:20, CG:20	30% MIP, 3 set/10 rep, +2% MIP increase, twice/d, 8 days	Chest physiotherapy, mobilization	MIP, A-a gradient	MIP pre and post 8th day *p* < 0.05 A-a gradient pre NS-post 8th day *p* < 0.05 SpO_2_ pre NS-8th day *p* < 0.05
Elmarakby 2016 [17] USA	Before–After CABG IG:17, CG:16	30% MIP, 30 breaths Increase 2 cm H_2_O twice/d	Chest physiotherapy	MIP, A-a gradients	*p* < 0.0001
Miozzo et al., 2018 [18] Brazil	After CABG IG:13, CG:11	50% up to 80% MIP, 5 set/10 rep for 12 weeks + aerobic exercise	Aerobic exercise	MIP	NS
Zanini et al., 2019 [19] Brazil	After CABG 4 groups: 10 patients per group	G1: IMT (8 sets of 10 breaths/20% of MIP) + chest physiotherapy + exercise/mobilization G3: IMT + chest physiotherapy	G2: chest physiotherapy + exercise/mobilization G4: chest physiotherapy	MIP, 6MWDT, FEV_1_% pred, FVC% pred	6MWDT: *p* < 0.05 MIP: NS FEV_1_% pred: NS FVC% pred: NS
Dos Santos et al., 2019 [20] Brazil	After CABG IG:12, CG:12	IMT + resistance and aerobic training IMT: 50% MIP, 5 set/10 rep, up to 80% MIP at 8th week	Sham IMT + resistance and aerobic training Sham IMT (9 cm H_2_O)	MIP, VO_2_ peak, 6MWDT	VO_2_ peak and 6MWT: *p* < 0.05
Cargnin et al., 2019 [21] Brazil	After heart valve replacement IG:12, CG:13	30% MIP, 30 cycles, twice/d, 7 d/w, 4 weeks	Sham (9 cm H_2_O)	MIP, 6MWDT, FEV_1_, FVC	MIP: *p* < 0.05, 6MWDT: *p* < 0.05
Chen et al., 2019 [22] China	After heart surgery (CABG, Valve) IG:98, CG:99	30% MIP for 20 min, twice/day, 5 d/w	Sham IMT (9% MIP)	MIP, FEV_1_% pred, FVC% pred-hospitalization length	MIP, FEV_1_% pred, FVC% pred: *p* < 0.05, less hospitalization: *p* < 0.05
Cordeiro et al., 2020 [23] Brazil	After CABG IG:21, CG:21	IMT based on anerobic threshold from 10% MIP up to 80% MIP	40% MIP, 3 set/15 rep, twice/daily until hospital discharge	MIP, 6MWDT	NS
Fortes et al., 2021 [24] Brazil	After heart surgery (CABG, Valve) IG:15, CG:15	30% MIP, 30 cycles, twice/day for six days	Chest physiotherapy, mobilization	MIP, Peak Insp Flow	MIP: NS PIF: *p* < 0.05 at 6th postoperative day
Hegazy et al., 2021 [25] Egypt	After mitral valve replacement surgery IG:50, CG:50	40% MIP, +5–10% MIP, 6 set of 5 breaths, twice/daily until hospital discharge-up to 80% at 8th week	Chest physiotherapy, mobilization	MIP, 6MWDT, FEV_1_, FVC	*p* < 0.001

MIP: Maximal Inspiratory Pressure, MEP: Maximal Expiratory Pressure, IG: intervention group, CG: control group, 6MWDT: 6-Minute Walk Distance Test, PIF: Peak Inspiratory Flow, FEV_1_: Forced Expiratory Volume at 1 s, FVC: Forced Vital Capacity, TV: Tidal Volume, VC: Vital Capacity, TLC: Total Lung Capacity, MRC: Medical Research Council, IMT: Inspiratory Muscle Training, CABG: coronary artery bypass, rep: repetitions, NS: non-significant.

**Table 2:** PEDro quality assessment of studies.

Study	1	2	3	4	5	6	7	8	9	10	11	
Matheus et al., 2012 [11]	x			x				x		x	x	4/10
Kodric et al., 2013 [12]	x	x	x	x	x					x	x	6/10
Sobrinho et al., 2014 [13]	x	x	x	x						x	x	5/10
Hermes et al., 2015 [14]	x	x		x			x	x		x	x	6/10
Cordeiro et al., 2016 [15]	x			x					x	x	x	4/10
Turky et al., 2017 [16]	x	x		x				x		x	x	5/10
Elmarakby 2017 [17]	x	x	x	x						x	x	5/10
Miozzo et al., 2018 [18]	x	x	x	x						x	x	5/10
Zanini et al., 2019 [19]	x	x	x	x			x	x		x	x	7/10
Dos Santos et al., 2019 [20]	x	x	x	x			x			x	x	6/10
Cargnin et al., 2019 [21]	x	x		x	x		x	x	x	x	x	8/10
Chen et al., 2019 [22]	x	x	x	x			x			x	x	6/10
Cordeiro et al., 2020 [23]	x	x		x	x					x	x	5/10
Fortes et al., 2021 [24]	x	x		x			x			x	x	5/10
Hegazy et al., 2021 [25]	x	x	x	x			x	x	x	x	x	8/10
Total mean score												5.7

## Data Availability

Not applicable.
